# Development of Japanese Cancer Intelligence Quotient to Measure Cancer Literacy and Knowledge among Japanese Laypersons

**DOI:** 10.31662/jmaj.2022-0084

**Published:** 2022-09-26

**Authors:** Masanari Minamitani, Tomoya Mukai, Hideomi Yamashita, Atsuto Katano, Mitsunori Miyashita, Keiichi Nakagawa

**Affiliations:** 1 Department of Comprehensive Radiation Oncology, The University of Tokyo, Tokyo, Japan; 2 Graduate Schools for Law and Politics, The University of Tokyo, Tokyo, Japan; 3 Department of Radiology, The University of Tokyo Hospital, Tokyo, Japan; 4 Department of Palliative Nursing, Health Sciences, Tohoku University Graduate School of Medicine, Miyagi, Japan

**Keywords:** cancer prevention, health literacy, health promotion, Japan, web survey

## Abstract

**Introduction:**

Health literacy has been identified an essential factor in leading a healthy lifestyle. Because some cancer prevention and screening methods have been established, we believe that identifying disadvantaged populations with low literacy regarding cancer is crucial. Thus, in this study, we aim to create a self-administered cancer-specific health literacy scale to be administered to Japanese laypersons.

**Methods:**

Using definitions from previous studies, we constructed a scale named the Japanese Cancer Intelligence Quotient (JCIQ) for both literacy (JCIQ-L) and knowledge (JCIQ-K) aspects. We generated potential items for both aspects, extracted appropriate ones using two-step online surveys, and compared the JCIQ and cancer-preventive behaviors and cancer-screening intentions, both of which we set as alternative indicators of the right attitude and practice toward cancer by performing a multiple regression analysis from another web survey.

**Results:**

Between April and May 2020, we conducted three-step surveys online. After conducting the two-step surveys for thousands of people, we extracted 12 literacy questions and 22 knowledge questions using factor analysis and the correct answer ratio of every item. In the final investigation of 3,094 people, a multiple regression analysis found that the JCIQ-L and JCIQ-K were significant factors in terms of predicting both behaviors (JCIQ-L:β = 0.07, *p* < 0.001, JCIQ-K:β = 0.05, *p* < 0.01) and willingness (JCIQ-L:β = 0.04, *p* < 0.05, JCIQ-K:β = 0.17, *p* < 0.001) after adjusting for participant characteristics (e.g., gender, age, income level, employment status).

**Conclusions:**

We developed the first reliable scale for measuring cancer literacy and knowledge of Japanese laypersons.

## Introduction

Cancer is known to be a severe global disease burden. In 2020, 19.3 million people were reported to have been diagnosed with cancer, and 10.0 million died from this disease worldwide ^(1)^. This number is expected to increase in the future ^(1)^. In fact, the Japanese National Cancer Center has estimated that two-thirds of males and one-half of females will suffer from cancer in their lifetime ^(2), (3)^. About half of cancer incidence and mortality can be prevented by modifying behavior, living environment, vaccination, and early detection through screening ^(4), (5), (6)^. The Ministry of Health, Labor, and Welfare in Japan recommends improving lifestyle factors such as cigarette smoking, alcohol consumption, diet, physical activity, and relative weight (body mass index [BMI]) for cancer prevention, in addition to checking the status of infection ^(3), (6)^. People with low health literacy have been determined to have a higher risk of cancer incidence; therefore, developing knowledge and health literacy to implement healthy lifestyles could lead to cancer prevention ^(7), (8), (9)^.

Health literacy is known as “the personal characteristics and social resources needed for individuals and communities to access, understand, appraise and use information and services to make decisions about health ^(10)^.” Literacy is essential to improve health and reduce health inequities, but it has been underestimated in the field of cancer ^(11), (12), (13)^. Given some reliable prevention and screening methods in oncology, health literacy concerning cancer among ordinary people, not cancer patients, is deemed crucial; therefore, a scale measuring literacy would be a big help to identify disadvantaged populations and provide interventions.

The European Health Literacy Survey Questionnaire (HLS-EU-Q47) is a well-known comprehensive health literacy measurement developed by Sørensen from a conceptual model composed of 12 subdimensions that consist of health-related competencies to access, understand, appraise, and apply the information within three domains (healthcare, disease prevention, and health promotion) ^(14), (15)^. The Japanese version of the HLS-EU-Q47, the J-HLS-EU-Q47, was available, which allows respondents to answer in a self-administered format ^(16)^. The short versions of the HLS-EU-Q47 with 16 or 12 items, that is, the HLS-EU-Q16, HLS-Q12, and HL-SF12, have also been developed ^(17), (18), (19)^. On the contrary, several cancer-specific literacy measurements were developed, although most were designed to measure cancer literacy for cancer patients ^(20), (21), (22)^. As a measurement of cancer-specific literacy for laypersons, the Cancer Literacy Score (CLS) was developed by Diviani under the following definition: “all the knowledge a layperson needs to possess to understand the information and advice the health system has to offer with regard to preventing, diagnosing and treating cancer ^(23), (24)^.” This definition was based on the hypothesis that specific cancer-related health outcomes can be better predicted by the CLS than by general health literacy alone, as abundant health literacy does not necessarily mean very literate for a specific condition such as cancer ^(23), (24)^. A previous study has reported the association between the CLS and cancer-related behavior ^(25)^. However, this measurement consisted of the orally administered Italian/English-language questionnaire and was not self-administered.

Thus, in this study, we aim to create a self-administered cancer-specific health literacy scale for Japanese laypersons, to assess both aspects of literacy and knowledge, applying the HLS-EU definition of health literacy into the field of cancer and using the definition of the CLS for cancer knowledge. Moreover, we aimed to validate the scale by examining its relationship with cancer-related behaviors, to use the scale when determining the effectiveness of health education about cancer, and to identify disadvantaged people in the future.

## Materials and Methods

### Development of the Japanese Cancer Intelligence Quotient

#### Applying the cancer literacy concept

**Figure 1.**
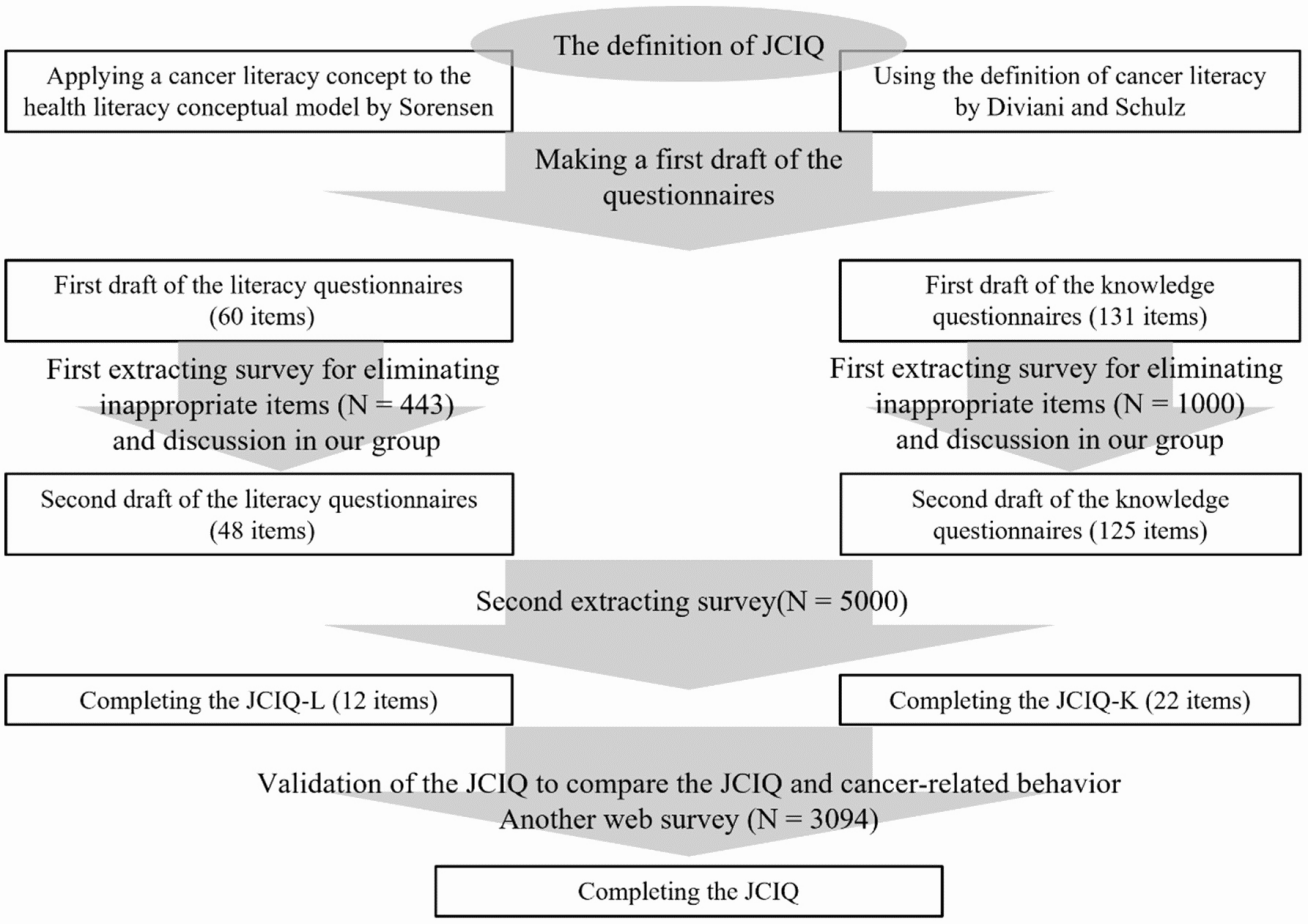
Flow of completing the scale. Abbreviations: JCIQ, Japanese Cancer Intelligence Quotient; JCIQ-L, Japanese Cancer Intelligence Quotient-Literacy; JCIQ-K, Japanese Cancer Intelligence Quotient-Knowledge.

We created the Japanese Cancer Intelligence Quotient (JCIQ), which consisted of a literacy aspect (JCIQ-L) and a knowledge one (JCIQ-K) in Japanese laypersons. In this paper, we defined the Japanese laypersons as people aged between 20 and 70 years old, excluding cancer patients and survivors. For the literacy aspect, we applied the conceptual model developed by Sørensen to oncology as follows: Cancer literacy is linked to literacy and entails the motivation, knowledge, and competencies to access, understand, appraise, and apply cancer information to make judgments and decisions in everyday life concerning healthcare with regard to cancer, cancer prevention, and health promotion about cancer to maintain or improve quality of life throughout the course of life ^(14)^. Meanwhile, for the knowledge aspect, the conceptual model of Diviani was used directly, as described above ^(23)^. Figure 1 presents the details of the development of the JCIQ.

#### Item generation

Our group drafted the first questionnaires of the JCIQ. The draft of the JCIQ-L consisted of 60 questions, 5 items for each of the 12 subsections, derived from the J-HLS-EU-Q47 ^(16)^. These answers were categorized into five options: a 4-point Likert-type scale (1 = very difficult, 2 = fairly difficult, 3 = fairly easy, 4 = very easy) and one “don’t know/not applicable” option, which was coded as a missing value. We created 131 draft questions on the JCIQ-K for the 5 subscales of the CLS: cancer risk, detection and diagnosis, treatment, coping with the disease, and information ^(23), (24)^. In preparing the candidates and correct answers of the JCIQ-K, we referred to previous works, including the CLS and the web pages of the “cancer information service” from the National Cancer Center in Japan, which is a popular cancer-informative site targeted to both cancer patients and the laypersons in Japan ^(1), (24), (26), (27), (28), (29)^. The answers of the JCIQ-K consisted of three options: “correct,” “incorrect,” and “don’t know.” The first questionnaires of the JCIQ are shown in Supplement 1.

#### Extracting surveys and participants

To extract the appropriate questions from the draft of the JCIQ, we conducted a web-based anonymous survey. We aimed to carry out the first step by extracting the survey to 500 participants for the JCIQ-L and 1,000 for the JCIQ-K. We then revised or removed draft questions with unclear meanings from the “don't know” rate in each questionnaire. The targeted population for the second step of extracting questions was 5,000 people aged between 20 and 80 years, excluding cancer patients and survivors. After reading the purpose, the participants consented to the survey and continued responding to the questions. Respondents received some cashable reward points, nearly 100 Japanese yen, after participating in this study. We then performed a factor analysis and chose 12 questions from each subsection of the JCIQ-L with the largest factor loadings after comparing those among each subscale. Thereafter, we carried out confirmatory factor analysis using the four factors for each domain of healthcare, disease prevention, and health promotion. We calculated the comparative fit index (CFI) and the root mean square error of approximation (RMSEA). A CFI value of 0.90 or higher is generally considered to be acceptable for the model; an RMSEA value of less than 0.05 represents a good fit, and a value of less than 0.08 is within acceptable limits. For the JCIQ-K, we extracted about 20 questions in total for each of the 5 subscales using the correct answer ratio by every item considering the usefulness of the JCIQ. To discriminate between higher and lower levels of knowledge, questions with a correct answer ratio of about the 25th and 75th percentile were selected for each subscale. After combining the JCIQ-L with the JCIQ-K, we completed the extraction of the JCIQ.

### Validation of the JCIQ

#### Development of the questionnaire

After the development of the JCIQ, another web survey was conducted to test the validity of the JCIQ to measure cancer literacy by comparing the association between the JCIQ and cancer-preventive behaviors and cancer-screening intentions. We regarded these two activities as alternative indicators of right attitudes and practices toward cancer, in reference to a previous study ^(25)^. The survey instrument included questions on five lifestyle habits that are known to be essential factors in preventing cancer, as referenced in the National Health and Nutrition Survey in Japan ^(6), (30), (31), (32), (33)^. In addition, we included the willingness to undergo cancer screening in the future in addition to the JCIQ and sociodemographic background, such as gender, age, marital status, number of children, annual income level, education, and employment status.

#### Participants

We included individuals between 20 and 70 years old and conducted the survey according to the population age distribution of the 2015 national census, with an upper limit at <70 years of age ^(34)^. We excluded those with a history of cancer diagnosis and individuals who participated in the extraction study.

#### Statistical analysis

The JCIQ-L index scores were standardized on a scale from 0 to 50, as in previous studies, using the following formula: (MEAN − 1) × (50/3), where MEAN represents the average score of all responses for each participant ^(16)^. The JCIQ-K scores were calculated by halving the proportion of correct answers to whole JCIQ-K questions. Through these processes, each score was on a 50-point scale. We considered as invalid and excluded any participants with more than 20% of responses of “don’t know/not applicable” for the JCIQ-L or who answered “don’t know” for all options of the JCIQ-K. Next, we defined each cancer-preventive lifestyle as follows: Current smokers and ex-smokers were considered “smokers,” in contrast to “never smokers,” who had never smoked. “Moderate drinkers” referred to individuals who do not drink or consume <140 g of ethanol per week or as “excessive drinkers” otherwise. Respondents who ate six or more small bowls of vegetables and fruits per day were labeled “adequate vegetables,” whereas those who consume less were “inadequate vegetables.” People with “adequate exercise” engaged in more than 30 minutes of activity at least two times per week and as “inadequate exercise” otherwise. Participants with a BMI ≥25 kg/m^2^ were classified with “obesity.” Individuals were considered “appropriate weight” if their BMI was <25. Criteria for alcohol intake, vegetable intake, exercise, and obesity used the cutoffs from the National Health and Nutrition Survey ^(33)^. For the analysis, we created the cancer-preventive score by allocating a value of 1 to healthy responses and a value of 0 to unhealthy ones and totaling the values of the five items. Respondents’ intentions to adhere to cancer screening was rated a continuous variable of a 5-point Likert-type scale.

We used a chi-square test for categorical variables and a *t*-test for continuous variables. We used a t-test to compare both JCIQ scores with the participants’ characteristics and cancer-related behaviors. Each score was presented as mean ± standard deviation. We then dichotomized categorical variables, with ages and income levels determined by a median split technique and with employment status divided by whether the participant had a job or not. We conducted a multiple regression analysis to examine the contributions of the JCIQ-L and JCIQ-K in explaining variance in their cancer-preventive scores or their cancer-screening intentions, which we set as an alternate benchmark for appropriate attitudes and practices against problems about cancer, as has been mentioned previously ^(25)^. In the regression model, each JCIQ index and the sociodemographic characteristics were added as potential confounders, as they were linked to health-related behaviors ^(6), (35), (36)^. All analyses were conducted using IBM SPSS for Windows, version 27.0. A two-sided *p*-value of less than 0.05 was considered statistically significant.

This study received approval from the Institutional Review Board of the Graduate School of Medicine and Faculty of Medicine, the University of Tokyo (2019363NI).

## Results

### Study sample characteristics

We conducted three-step surveys online between April and May 2020. Table 1 presents the demographic and clinical characteristics of each survey. Before the target numbers were met, we excluded 371 participants from the second extracting survey and 226 from the validation survey, respectively, due to their history of cancer diagnosis. The ratio of males to females was noted to be roughly equal in each survey, with few children or elderly people older than 70 included. In the validation study, half of the respondents had a college degree or higher, and >70% were workers. No significant differences were identified between participants and samples, except for vegetable intake (*p* < 0.001) and willingness to undergo cancer screening (*p* < 0.01).

**Table 1.** Baseline Characteristics of the Participants in the Extracting Surveys and the Validation Survey.

	First extracting survey				Second extracting survey		Validation survey				
	JCIQ-L (N = 443)		JCIQ-K (N = 1000)		(N = 5000)		Total participants (N = 3094)		Participants for analysis (N = 2454)		
	N	%	N	%	N	%	N	%	N	%	*P*-value
Gender											0.81
Male	233	53%	494	49%	2468	49%	1547	50%	1218	50%	
Female	210	47%	506	51%	2532	51%	1547	50%	1236	50%	
Age group (year)											0.96
≤19	5	1%	0	0%	0	0%	0	0%	0	0%	
20-29	19	4%	132	13%	658	13%	479	15%	397	16%	
30-39	64	14%	166	17%	832	17%	604	20%	477	19%	
40-49	137	31%	196	20%	980	20%	712	23%	553	23%	
50-59	105	24%	164	16%	822	16%	598	19%	466	19%	
60-69	80	18%	193	19%	964	19%	701	23%	561	23%	
70-79	27	6%	149	15%	744	15%	0	0%	0	0%	
80≥	6	1%	0	0%	0	0%	0	0%	0	0%	
Marital status											0.46
Single/widowed/div					1874	37%	1204	39%	930	38%	
Married					3126	63%	1890	61%	1524	62%	
Number of children											0.54
Zero					2050	41%	1406	45%	1094	45%	
Any					2950	59%	1688	55%	1360	55%	
Income level											0.13
< JPY2,000,000					1759	35%	1127	36%	901	37%	
JPY2,000,000-JPY3,999,999					1131	23%	699	23%	567	23%	
JPY4,000,000-JPY5,999,999					682	14%	490	16%	409	17%	
JPY6,000,000-JPY9,999,999					423	8%	296	10%	246	10%	
JPY10,000,000>					103	2%	9	3%	79	3%	
Unknown/not answer					902	18%	393	13%	252	10%	
Education											0.052
High school or less							1677	54%	1265	52%	
College or more							1417	46%	1189	48%	
Employment status											0.47
Self-employed worker							239	8%	190	8%	
Non-regularly employed worker							539	17%	413	17%	
Wage & salary worker							1450	47%	1200	49%	
Jobless							866	28%	651	27%	
Smoking											0.60
Never smoker							1880	61%	1522	62%	
Past smoker (smokers)							626	20%	487	20%	
Current smoker (smokers)							588	19%	445	18%	
Alcohol										0.80
Moderate drinker							2487	80%	1980	81%	
Excessive drinker							607	20%	474	19%	
Vegetables											<0.001
Adequate vegetables							688	22%	382	16%	
Inadequate vegetables							2406	78%	2072	84%	
Exercise											0.35
Adequate exercise							808	26%	669	27%	
Inadequate exercise							2286	74%	1785	73%	
BMI											0.87
<25 (appropriate weight)							2448	79%	1947	79%	
≥25 (obesity)							646	21%	507	21%	
Intentions for cancer screening											<0.01
Very high							650	21%	572	23%	
Moderately high							1168	38%	975	40%	
Intermediate							949	31%	680	28%	
Moderately low							259	8%	196	8%	
Very low							68	2%	31	1%	

Abbreviations: JCIQ-L, Japanese Cancer Intelligence Quotient-Literacy; JCIQ-K, Japanese Cancer Intelligence Quotient-Knowledge; JPY, Japanese Yen; BMI, body mass index Data are presented as the number of subjects in each group with percentages.

### Development of the JCIQ

Supplement 1 provides the list of candidate questions and answers. In the first-step extracting survey, 12 items were dropped and 13 were revised in the JCIQ-L, as 13 were dropped and 7 were revised in the JCIQ-K. In the second-step extracting survey, 12 items with the most prominent factor loadings in each section were extracted in the JCIQ-L, and the CFI and RMSEA values were 0.934 and 0.060, respectively. In the JCIQ-K, eight items from cancer risk, six from detection and diagnosis, four from treatment, two from coping with the disease, and two from information were selected. Table 2 lists the 34 questions in the JCIQ.

**Table 2.** Percentage of Respondents Giving Each Response on the JCIQ at the Validation Survey (Translated into English).

			Items	Very easy	Fairly easy	Fairly difficult	Very difficult	Don’t know/not applicable
JCIQ-L							
Healthcare	Access	Q1	Finding out what to do in case of cancer suspected	4%	21%	43%	28%	3%
	Understand	Q2	Understanding how to deal with cancer	3%	16%	49%	31%	1%
	Appraise	Q3	Judging the advantages and disadvantages when some cancer treatments are available	3%	11%	46%	39%	1%
	Apply	Q4	Identifying opinions of people around you and leading a lifestyle to prevent cancer occurrence	3%	23%	47%	22%	5%
Prevention	Access	Q5	Finding out how to undergo cancer screenings	17%	50%	24%	7%	1%
	Understand	Q6	Understanding what behaviors and lifestyles increase a cancer risk	8%	45%	36%	11%	1%
	Appraise	Q7	Judging the advantages and disadvantages of cancer screenings	4%	27%	48%	18%	3%
	Apply	Q8	Avoiding lifestyles that increase a cancer risk based on your knowledge	4%	35%	45%	15%	1%
Promotion	Access	Q9	Finding information on how your local government deals with cancer preventions	9%	44%	33%	10%	4%
	Understand	Q10	Understanding what political changes affect cancer preventions	4%	28%	48%	16%	4%
	Appraise	Q11	Judging what initiatives for cancer prevention from your local government are appropriate	3%	21%	53%	19%	5%
	Apply	Q12	Sharing correct cancer knowledge and prevention methods with friends and people around you	4%	21%	47%	27%	2%
JCIQ-K							
				**Correct**	**Incorrect**	**Don’t know**		
	Cancer risk	q1	Ingesting burnt food increases the chances of getting cancer.	59%	**23%**	18%		
		q2	HPV (human papillomavirus) infections increase the chances of getting cancer.	**35%**	5%	60%		
		q3	Asbestos exposure increases the chances of getting cancer.	**84%**	4%	12%		
		q4	Smoking increases the chances of getting lung cancers three to five more times.	**94%**	3%	3%		
		q5	Passive smoking does not increase the chances of getting lung cancer.	4%	**91%**	5%		
		q6	Adherence to healthy lifestyle habits can prevent nearly 90% of cancers.	30%	**28%**	42%		
		q7	To prevent cancer, you should achieve a BMI of 18.	8%	**45%**	47%		
		q8	Cancer could be inherited among some families.	**87%**	4%	9%		
	Detection and diagnosis	q9	Breast cancer screening is included in the cancer screenings recommended by the government.	11%	**63%**	26%		
		q10	Pancreatic cancer screening is included in the cancer screenings recommended by the government.	24%	**34%**	42%		
		q11	Sputum examination is recommended as a lung cancer screening for smokers by the government.	**77%**	6%	17%		
		q12	An annual examination starting at age 20 years consisting of mammography and breast ultrasound is recommended as breast cancer screening by the government.	18%	**34%**	47%		
		q13	The benefit of cancer screening is early detection and early intervention to suspected lesions.	**32%**	12%	56%		
		q14	Cancer screening can detect any small lesions.	40%	**27%**	33%		
	Treatment	q15	The three main cancer treatments consist of surgery, chemotherapy, and immunotherapy.	**79%**	5%	16%		
		q16	Cancer is not completely cured by radiotherapy.	8%	**73%**	19%
		q17	Patients should discuss with their doctors when making decisions about their cancer treatments.	36%	**23%**	42%		
		q18	Second opinions are not recommended in oncological fields because consulting for opinions requires time and cost.	32%	**28%**	40%		
	Coping with disease	q19	Occupational healthcare services are available to every employee when they are diagnosed or suspected of cancer.	**87%**	3%	10%		
		q20	Medical information is private and cannot be generally divulged to others without permission	3%	**79%**	19%		
	Information	q21	Cancer information from families is more trustworthy than from doctors because families are more kind.	**42%**	10%	49%		
		q22	Cancer information from medical guidelines is as trustworthy as from TVs and radios because each has its advantage.	**83%**	4%	14%		

Abbreviations: JCIQ-L, Japanese Cancer Intelligence Quotient-Literacy; JCIQ-K, Japanese Cancer Intelligence Quotient-Knowledge Correct answer choices of the JCIQ-K are shown in bold.

### Validation of the JCIQ

Table 2 presents the results of the participants’ responses to the JCIQ in the validation phase. The percentage of each “don’t know/not applicable” responses for the JCIQ-L was less than 5%. The average rate of correct answers was 57% for the JCIQ-K; the correct answers are marked in bold in Table 2. Table 3 shows the relationship between the JCIQ scores and participants’ characteristics, cancer-prevention behaviors, and intentions to undergo cancer screenings. Women (JCIQ-L: *p* = 0.04; JCIQ-K: *p* < 0.001) and elderly individuals (JCIQ-L: *p* = 0.03; JCIQ-K: *p* < 0.001) had significantly higher scores, and no significant differences were noted in terms of income level (JCIQ-L: *p* = 0.61; JCIQ-K: *p* = 0.26), education (JCIQ-L: *p* = 0.15; JCIQ-K: *p* = 0.29), or employment status (JCIQ-L: *p* = 0.09; JCIQ-K: *p* = 0.08). Those with healthier behaviors in terms of smoking (JCIQ-L: 20.2 vs. 19.5, *p* < 0.05; JCIQ-K: 28.8 vs. 27.5, *p* < 0.001) and vegetable intake (JCIQ-L: 21.2 vs. 19.7, *p* < 0.01; JCIQ-K: 29.2 vs. 28.2, *p* < 0.05) scored significantly higher, whereas those who reported exercising frequently had a higher JCIQ-L score (21.0 vs. 19.6, *p* < 0.001). In addition, the group with a higher willingness to undergo screenings also scored higher (JCIQ-L: 20.6 vs. 18.8, *p* < 0.001; JCIQ-K: 29.5 vs. 26.4, *p* < 0.001).

**Table 3.** Relationships between the Participants’ JCIQ Score and Characteristics.

		JCIQ-L				JCIQ-K			
		Mean	SD	*P*-value	Cohen’s d	Mean	SD	*P*-value	Cohen’s d
Gender				0.04	−0.08			<0.001	−0.22
	Male	19.6	8.7			27.4	8.9		
	Female	20.3	8.1			29.3	7.9		
Age group (year)				0.03	−0.09			<0.001	−0.20
	≤49	19.7	8.4			27.7	8.9		
	>50	20.4	8.3			29.3	7.7		
Marital status				0.70	−0.02			<0.01	−0.23
	Single/widowed/divorced	19.9	8.4			27.2	8.8		
	Married	20.0	8.4			29.1	8.1		
Number of children				0.59	−0.02			<0.001	−0.21
	Zero	19.9	8.4			27.8	8.8	
	Any	20.0	8.4			29.1	8.0		
Income level				0.61	0.02			0.26	−0.05
	< JPY2,000,000 and unknown/no answer	20.0	8.3			28.1	8.4		
	JPY2,000,000-	19.9	8.5			28.5	8.4		
Education				0.15	−0.01			0.29	−0.20
	High school or less	19.9	8.2			27.5	8.5		
	College or more	20.0	8.6			29.2	8.3		
Employment status				0.09	−0.08			0.08	−0.08
	Worker	19.8	8.3			28.2	8.6		
	Jobless	20.4	8.5			28.8	7.9		
Smoking				0.04	0.08			<0.001	0.15
	Never smoker	20.2	8.4			28.8	8.4		
	Smoker	19.5	8.4			27.5	8.5		
Alcohol				0.54	0.03			0.53	0.03
	Moderate drinker	20.0	8.3			28.4	8.4		
	Excessive drinker	19.7	8.6			28.1	8.5		
Vegetables				<0.01	0.18			0.03	0.12
	Adequate vegetables	21.2	8.9			29.2	8.3		
	Inadequate vegetables	19.7	8.3			28.2	8.4		
Exercise				<0.001	0.17			0.19	0.06
	Adequate exercise	21.0	8.3			28.7	8.5		
	Inadequate exercise	19.6	8.4			28.2	8.4		
BMI				0.15	0.07			0.29	0.05
	<25 (appropriate weight)	20.1	8.3			28.4	8.4		
	≥25 (obesity)	19.5	8.9			28.0	8.5		
Cancer-preventive score				<0.001	0.13		<0.01	0.12
	3-5	20.4	8.3			28.7	8.4		
	0-2	19.3	8.5			27.8	8.4		
Intentions for cancer screening				<0.001	0.22			<0.001	0.38
	Very high, moderately high	20.6	8.4			29.5	8.1		
	Intermediate, moderately low, very low	18.8	8.4			26.4	8.6		

Abbreviations: JCIQ-L, Japanese Cancer Intelligence Quotient-Literacy; JCIQ-K, Japanese Cancer Intelligence Quotient-Knowledge; JPY, Japanese yen; BMI, body mass index

Table 4 describes the results of the multiple regression analysis, in which dependent variables were the cancer-preventive score and intentions to undergo cancer screening. For cancer prevention, the JCIQ-L (β = 0.07, *p* < 0.001), JCIQ-K (β = 0.05, *p* < 0.01), gender (β = −0.25, *p* < 0.001), and age (β = −0.17, *p* < 0.001) were found to be significantly associated. For screening intention, the JCIQ-L (β = 0.04, *p* < 0.05), JCIQ-K (β = 0.17, *p* < 0.001), gender (β = −0.12, *p* < 0.001), and income level (β = −0.09, *p* < 0.001) were independent predictors. These two models suggest that a high JCIQ score and being female were positively effective on the benchmark that we set as the right attitude and practice toward cancer among the Japanese laypersons.

**Table 4.** Multiple Regression Analysis with the Cancer-Preventive Score and Cancer-Screening Intention as Dependent Variable, Predicted by Participants’ Characteristics and the JCIQ.

		Cancer-preventive score								Cancer-screening intention						
		*B*-value	SE	*B*-value		*R^2^* value	*P*-value	Adjusted *R^2^*		*B*-value	SE	*B*-value		*R^2^* value	*P*-value	Adjusted *R^2^*
Variable						0.12	<0.001	0.11						0.06	<0.001	0.05
	Gender	−0.50	0.04	−0.25	***					−0.23	0.04	−0.12	***			
	Age	−0.01	0.00	−0.17	***					0.00	0.00	0.02				
	Income level	0.06	0.05	0.03						−0.18	0.05	−0.09	***			
	Employment status	−0.09	0.05	−0.04						0.08	0.05	0.04				
	JCIQ-L	0.01	0.00	0.07	***					0.00	0.00	0.04	*			
	JCIQ-K	0.01	0.00	0.05	**					0.02	0.00	0.17	***			

Abbreviations: JCIQ, Japanese Cancer Intelligence Quotient; JCIQ-L, Japanese Cancer Intelligence Quotient-Literacy; JCIQ-K, Japanese Cancer Intelligence Quotient-Knowledge; SE, standard error; *p < 0.05; **p < 0.01; ***p < 0.001.

## Discussion

In this study, we aimed to create a cancer literacy scale that could be available in Japan by applying the definitions used in previous research. We demonstrated through multiple regression analysis that our scale was positively associated with cancer-related health behaviors and willingness to undergo cancer screening. Thus, our JCIQ score is potentially valuable in terms of identifying disadvantaged populations that should be aware of cancer-related matters.

Using health and cancer literacy concepts from previous studies, we generated the first JCIQ. Although applying the HLS-EU concepts to cancer literacy might be controversial, health literacy skills are indispensable in cancer approaches; thus, their use should be tolerated ^(7), (9), (14)^. Previously reported measurements for cancer patients are inapplicable to laypersons, as people who have been diagnosed with cancer drastically would change their awareness and thoughts toward the disease ^(20), (21), (22)^. The CLS is an example for healthy individuals, but it involves face-to-face interaction and open questions ^(24)^. The use of the self-administered JCIQ is more valuable in efficiently administering extensive surveys to identify disadvantaged people. To the best of our knowledge, this is the first attempt to create a scale to measure cancer awareness in terms of both literacy and knowledge.

To assess the feasibility of the JCIQ, we evaluated whether the scale is associated with healthy behaviors that avoid the risk of cancer and cancer-screening uptake. In the univariate analysis, comparisons of the two groups concerning alcohol consumption, BMI, and exercise showed no significant differences in one or both of the two scores of the JCIQ, although the scores were noted to be higher in each healthy group. However, because domestic investigations showed that combining the five healthy habits can reduce cancer risk, it is reasonable to sum the five at the analysis, including adequate smoking and diet lifestyle ^(6), (30)^. In the multiple regression analysis, being female, younger age, and higher income level exhibited a roughly positive trend in predictor variables. A previous Japanese study reported a difference in the screening uptake rate depending on employment status, but it varied by the size of the company ^(37)^. Having a job or not could contribute to the insignificant difference in dependent variables. Each JCIQ score was a significant factor in predicting behaviors and willingness. Interestingly, the literacy score was more strongly correlated with cancer-related health behaviors, whereas the coefficient for knowledge was found to be larger for willingness to undergo screening. Previous studies have also reported that knowledge has a greater impact than social status does, which is similar to the findings of this current study ^(25)^. Japanese populations face many oncological problems, such as lower cancer-screening rates than other developed countries, a human papillomavirus vaccination rate of ≤1%, insufficient tobacco control, and discrepancies between public perceptions and epidemiological facts for cancer prognosis and incidence ^(38), (39), (40), (41)^. Amid this challenging situation, it remains uncertain which populations have less knowledge and awareness of cancer. Therefore, we must identify who is vulnerable and what interventions are effective. We believe the self-administered JCIQ is highly versatile and suitable for large-scale surveys.

In this study, we have examined the association between the JCIQ and cancer-related behaviors. We could not indicate whether improvements in the JCIQ lead to improvements in healthy behaviors. The JCIQ, which was described in Supplement 1, comprised psychological factors and did not include any behaviors. Our results support the importance of the association between psychological factors and habitual behaviors shown in previous studies ^(42)^. Therefore, further studies need to elucidate whether health education can lead to increased literacy and knowledge and behavior change.

This study has some limitations. The first limitation of this study is that our research was based on a web-based survey. The participants who responded to the survey did not necessarily represent Japanese laypersons. There are several confounders and biases in web-based surveys, including digital literacy, computing hardware resources, and accessibility of the Internet ^(43)^. Second, we did not exclude participants with a family history of cancer at the extracting and validation survey. Family history might work as a confounder in our study. Third, in the validation phase, we selected explanatory variables following previous studies. However, unmeasured confounders could have possibly affected our multiple regression analysis. Fourth, we adopted cancer-preventive behaviors and cancer-screening intentions as alternatives of decent awareness and attitude toward cancer. Although these dependent variables were derived from a previous study and used as measurable parameters, they may not fully reflect behavioral differences based on the definitions of this study. Fifth, we could not deny that the selection of the JCIQ-K included some arbitrariness. We initially considered extracting candidates using item response theory, but the analysis resulted in revealing the multifactorial aspects of cancer knowledge. Validation showed both the JCIQ-K and JCIQ-L to be significant factors, but we could not guarantee that the items were necessarily appropriate, as we selected them based on the correct answer ratio of each question. Next, we did not compare the JCIQ with previously reported scores, which should be considered in future research. Finally, the JCIQ was intended for current use in Japan, and the correctness or incorrectness of the items may change in the future and require revision. The recommended age and contents of cancer screenings could easily change according to the results of clinical trials. Despite these limitations, our study contributes important insights.

This study demonstrates the creation of a scale for measuring cancer literacy in healthy Japanese laypeople. The JCIQ was self-administered, written in Japanese, and conveniently used in many situations. Although this scale needs further revision and improvement, it can potentially identify disadvantaged people for cancer-related problems.

## Article Information

### Conflicts of Interest

The authors declare that there are no conflicts of interest. The Department of Comprehensive Radiation Oncology, to which Masanari Minamitani and Keiichi Nakagawa belong, is an endowment department, supported with an unrestricted grant from Elekta K. K. However, the sponsor had no role in this study.

### Sources of Funding

This work was supported by the *Corporate Action to Promote Cancer Control* entrusted by the Ministry of Health, Labour and Welfare. Keiichi Nakagawa is an advisory board member of the association.

### Author Contributions

Masanari Minamitani designed the study, analyzed the data, and wrote the results. Tomoya Mukai supported Masanari Minamitani in all aspects of this study. Hideomi Yamashita, Atsuto Katano, and Mitsunori Miyashita contributed to the draft manuscript preparation. Keiichi Nakagawa provided funding acquisition, study conception, and interpretation of the result. All authors read and approved the final manuscript.

### Approval by Institutional Review Board (IRB)

This study received approval from the Institutional Review Board of the Graduate School of Medicine and Faculty of Medicine, the University of Tokyo (2019363NI).

## Supplement

Supplement 1
