# Supplementary material for: Development of Japanese Cancer Intelligence Quotient to Measure Cancer Literacy and Knowledge among Japanese Laypersons
Source: JMA J. 2022 Sep 26;5(4):446–57. doi: 10.31662/jmaj.2022-0084 (PMC9646290; doi:10.31662/jmaj.2022-0084)
Supplement: Supplementary file 1 — Supplement 1 [file 2433-3298-5-4-0446-s001.pdf]

Supplement 1. Candidate items for JCIQ-L & JCIQ-K (translated into English)

		Items	1st survey	2nd survey
**JCIQ-L**				
Health care	Access	Finding out information about symptoms caused by cancer	✓	✓
		Finding out information about cancer treatments	✓	✓
		**Finding out what to do in case of cancer suspected**	✓	✓
		Finding out how to consult oncologists in case of cancer diagnosed	✓	
		Finding out how to consult oncologists in case of cancer suspected (revised for 2nd)		✓
		Finding out whom to ask for advice in case of cancer diagnosed	✓	
	Understand	Understanding information about cancer	✓	✓
		Understanding opinions from oncologists	✓	✓
		Understanding what to do in case of cancer diagnosed	✓	
		**Understanding how to deal with cancer** (revised for 2nd)		✓
		Understanding that symptoms caused by cancer wouldn't appear unless cancer advances	✓	
		Understanding symptoms caused by cancer (revised for 2nd)		✓
		Understanding that cancer treatments are not necessarily pre-determined in case of cancer diagnosed	✓	
	Appraise	Judging whether opinions of oncologists about cancer are correct or not	✓	✓
		Judging whether opinions of people around you about cancer are correct or not	✓	
		Judging whether information from mass media about cancer is trustworthy or not	✓	
		Judging whether information from TV and the Internet about cancer are trustworthy or not (revised for 2nd)		✓
		**Judging the advantages and disadvantages when some cancer treatments are available**	✓	✓
		Judging what treatments are appropriate for your cancer	✓	✓
	Apply	Choosing appropriate treatments for your cancer based on your knowledge in case of cancer diagnosed	✓	✓
		Communicating your desires to your doctor based on your knowledge in case of cancer diagnosed	✓	
		Taking care not to get cancer, based on oncologists' opinions	✓	
		Avoiding behaviors that increase the risk of cancer, based on your knowledge (revised for 2nd)		✓
		**Identifying opinions of people around you and leading a lifestyle to prevent cancer occurrence**		✓
		Refining your lifestyle based on information about cancer	✓	✓

Prevention	Access	Finding out information about a cancer risk	✓	
		Finding out what behaviors and lifestyles develop a cancer risk	✓	✓
		Finding out when and where to undergo recommended cancer screenings	✓	
		Finding out when and where to undergo cancer screenings (revised for 2nd)		✓
		**Finding out how to undergo cancer screenings**		✓
		Finding out information about cancer preventions	✓	✓
	Understand	Understanding what increases a cancer risk	✓	✓
		**Understanding what behaviors and lifestyles increase a cancer risk**	✓	✓
		Understanding that smoking, lack of exercise, and excessive alcohol consumption develop a cancer risk	✓	
		Understanding that smoking, lack of exercise, and excessive alcohol consumption cause cancer (revised for 2nd)		✓
		Understanding that cancer preventions such as the cervical cancer vaccine are necessary for children	✓	
		Understanding why cancer screenings are essential	✓	✓
	Appraise	Judging the reliability of whether smoking, lack of exercise, and excessive alcohol consumption cause cancer	✓	✓
		Judging the acceptability of vaccines for cancer preventions	✓	
		Judging whether you should undergo cancer screenings	✓	✓
		**Judging the advantages and disadvantages of cancer screenings**	✓	✓
		Judging what kind of cancer screenings you should undergo	✓	✓
	Apply	**Avoiding the lifestyles which increase a cancer risk based on your knowledge**	✓	✓
		Acting to protect yourself from getting cancer, based on the information from TV and the Internet	✓	✓
		Understanding what behaviors develop a cancer risk and avoiding such behaviors	✓	✓
		Judging the necessity of undergoing cancer screenings based on the information	✓	✓
		Judging whether to encourage children to get vaccinated for cancer preventions	✓	
Promotion	Access	Finding information on how you prevent cancer, such as adequate exercise and diet	✓	
		Finding information on how you prevent cancer, such as adequate exercise, diet and, living environment(revised for 2nd)		✓
		Finding information on what policies are implemented for cancer preventions	✓	
		Finding information on what policies the government implemented for cancer preventions (revised for 2nd)		✓
		Finding information on what initiatives are conduct for cancer preventions in your workplace	✓	
		Finding information on what initiatives are conduct for cancer preventions in your community	✓	

	**Finding information on how your local government deal with cancer preventions** (revised for 2nd)		✓	
	Finding information on what initiatives are conduct for cancer preventions around you	✓	✓	
Understand	Understanding why initiatives for cancer preventions in your community are necessary	✓	✓	
	Understanding the importance of exercise and activities for cancer preventions	✓		
	Understanding the importance of exercise, activities, and living environment for cancer preventions (revised for 2nd)		✓	
	**Understanding what political changes affect cancer preventions**	✓	✓	
	Understanding the importance of initiatives for cancer preventions in your workplace	✓		
	Understanding what initiatives of your local government decrease a cancer risk	✓	✓	
Appraise	Judging whether initiatives for cancer preventions around you are appropriate	✓	✓	
	Judging whether initiatives for cancer preventions in your workplace are appropriate	✓		
	**Judging what initiatives for cancer prevention from your local government are appropriate**	✓	✓	
	Judging what policies for cancer preventions increase and decrease a cancer risk	✓	✓	
	Judging whether information from mass media about cancer preventions are trustworthy	✓	✓	
Apply	Encouraging your workplace to deal with cancer preventions properly	✓		
	Participating in organizations that promote cancer preventions	✓		
	Sharing correct cancer knowledge and prevention methods with people around you (revised for 2nd)		✓	
	Sharing correct cancer knowledge and prevention methods through social media	✓		
	**Sharing correct cancer knowledge and prevention methods with friends and people around you** (revised for 2nd)		✓	
	Encouraging to prevent your community from increasing a cancer risk	✓	✓	
	Sharing correct cancer knowledge and prevention methods with your family	✓	✓	

**JCIQ-K**	**Items**			Answer
Cancer risk	Bruises and contusions increase the chances of getting cancer.	✓		I
	Contact with cancer patients increases the chances of getting cancer.	✓	✓	I

Wounds increase the chances of getting cancer.	✓		I
Smoking increases the chances of getting cancer.	✓	✓	C
Cell phone signals increase the chances of getting cancer.	✓		I
Exposure to the sun increases the chances of getting cancer.	✓		C
Obesity increases the chances of getting cancer.	✓	✓	C
Passive smoking increases the chances of getting cancer.	✓	✓	C
Excessive alcohol drinking increases the chances of getting cancer.	✓	✓	C
Lack of exercise increases the chances of getting cancer.	✓	✓	C
**Ingesting burnt food increases the chances of getting cancer.**	✓	✓	I
Eating a lot of processed products, such as sausages and hams, increase the chances of getting cancer.	✓	✓	C
Eating a lot of vegetables increases the chances of getting cancer.	✓	✓	I
H. pylori infections increase the chances of getting cancer.	✓	✓	C
**HPV (human papillomavirus) infections increase the chances of getting cancer.**	✓	✓	C
HCV (hepatitis C virus) and HBV (hepatitis B virus) infections increase the chances of getting cancer.	✓	✓	C
EBV (EB virus) infections increase the chances of getting cancer.	✓	✓	C
HIV infections increase the chances of getting cancer.	✓	✓	C
**Asbestos exposure increases the chances of getting cancer.**	✓	✓	C
Radiation increases the chances of getting cancer.	✓	✓	C
Getting older increases the chances of getting cancer.	✓	✓	C
Eating a diet with additives increases the chances of getting cancer. (revised for 2nd)		✓	I
Eating genetically modified foods increases the chances of getting cancer. (revised for 2nd)		✓	I
Eating microwaved food increases the chances of getting cancer. (revised for 2nd)		✓	I
Drinking beverages in plastic bottles increases the chances of getting cancer. (revised for 2nd)		✓	I
Feeling stressed increases the chances of getting cancer. (revised for 2nd)		✓	I

Living with Wi-Fi signals increases the chances of getting cancer. (revised for 2nd)		✓	I
Smoking is the preventable but leading cause of cancer in Japan.	✓	✓	C
**Smoking increases the chances of getting lung cancers three to five more times.**	✓	✓	C
Smoking increases the chances of getting lung cancers by nearly 30 times.	✓	✓	I
Smoking is linked to the development of bladder cancer.	✓	✓	C
Smoking is linked to the development of throat cancer.	✓	✓	C
Smoking is responsible for nearly 20% of cancer deaths worldwide.	✓		C
If you don’t smoke, you won’t get lung cancer.	✓	✓	I
Excessive sun exposure can lead to skin cancer.	✓		C
Obesity increases the chances of getting colorectal cancer.	✓	✓	C
Obesity increases the chances of getting liver cancer.	✓	✓	C
Obesity increases the chances of getting uterus cancer.	✓	✓	C
Becoming too slim does not increase increases the chances of getting cancer.	✓	✓	I
**Passive smoking does not increase the chances of getting lung cancer.**	✓	✓	I
Passive smoking from e-cigarettes does not increase the chances of getting lung cancer.	✓	✓	I
Excessive alcohol drinking increases the chances of getting throat cancer.	✓	✓	C
Eating and drinking hot foods/liquids increase the chances of getting esophagus cancer.	✓	✓	C
Eating a lot of processed meat increases the chances of getting colorectal cancer.	✓	✓	C
The carrier rate of H. pylori in Japan remains still high and is not decreasing.	✓	✓	I
Most cervical cancers are attributed to HPV (human papillomavirus) infection.	✓	✓	C
Most uterus cancers are attributed to HPV (human papillomavirus) infections.	✓	✓	I
Most liver cancers are attributed to virus infections.	✓	✓	C
Some throat cancers are attributed to virus infections.	✓	✓	C
HIV infections increase the chances of getting cervical cancer	✓	✓	C

**Adherence to healthy lifestyle habits can prevent nearly 90% of cancers.**	✓	✓	I
Nearly half of all cancers are inevitable.	✓	✓	C
Not smoking, moderate alcohol consumption, a low-salt diet, exercise, and proper diet can reduce the chances of getting cancer by about 40%.	✓	✓	C
To prevent cancer, you should reduce the number of cigarettes you smoke by half.	✓	✓	I
To prevent cancer, you should switch to low-tar cigarettes.	✓	✓	I
To prevent cancer, you should stop smoking.	✓	✓	C
To prevent cancer, you should replace cigarettes with e-cigarettes or heat-not-burn tobacco products.	✓	✓	I
To prevent skin cancer, you should use appropriate sunscreens.	✓		C
To prevent cancer, you should maintain a desirable weight.	✓	✓	C
**To prevent cancer, you should achieve a BMI of 18.**	✓	✓	I
To prevent cancer, you should avoid cigarette smoke as much as possible.	✓	✓	C
To prevent cancer, you should limit alcohol consumption to no more than one beer per day.	✓	✓	C
To prevent cancer, you should engage in moderate exercise in your daily life.	✓	✓	C
To prevent cancer, you should eat a well-balanced diet.	✓	✓	C
To prevent cancer, you should reduce salt intake.	✓	✓	C
To prevent cancer, you should eradicate H. pylori as early as possible.	✓	✓	C
Vaccines can reduce the chance of getting some cancers.	✓	✓	C
To prevent cancer, you should try to have safe sex.	✓	✓	C
To prevent cancer, you should replace asbestos walls with other materials.	✓	✓	C
To prevent cancer, you should take supplements labeled cancer prevention.	✓	✓	I
To prevent cancer, you should drink a lot of water.	✓	✓	I
To prevent cancer, you should refrain from using cell phones.	✓	✓	I
To prevent cancer, you should take medicine.	✓		I

	Genetic cancers account for nearly 30% of all cancers	✓	✓	I
	**Cancer could be inherited among some families.**	✓	✓	C
	30% to 50% of all cancers worldwide are preventable.	✓	✓	C
Detection and diagnosis	Gastric cancer screening is included in the cancer screening recommended by the government.	✓	✓	C
	Colorectal cancer screening is included in the cancer screening recommended by the government.	✓	✓	C
	Lung cancer screening is included in the cancer screening recommended by the government.	✓	✓	C
	**Breast cancer screening is included in the cancer screenings recommended by the government.**	✓	✓	C
	Cervical cancer screening is included in the cancer screening recommended by the government.	✓	✓	C
	Prostate cancer screening is included in the cancer screening recommended by the government.	✓	✓	I
	**Pancreatic cancer screening is included in the cancer screenings recommended by the government.**	✓	✓	I
	Skin cancer screening is included in the cancer screening recommended by the government.	✓	✓	I
	Liver cancer screening is included in the cancer screening recommended by the government.	✓	✓	I
	Gastroscopy is recommended as a gastric cancer screening by the government.	✓	✓	C
	Barium examination of the stomach is recommended as a gastric cancer screening by the government.	✓	✓	C
	A fecal occult blood test is recommended as a colorectal cancer screening by the government.	✓	✓	C
	Colonoscopy is recommended as a colorectal cancer screening by the government.	✓	✓	I
	A chest x-ray is recommended as a lung cancer screening by the government.	✓	✓	C
	**Sputum examination is recommended as a lung cancer screening for smokers by the government.**	✓	✓	C
	A chest CT scan is recommended as a lung cancer screening by the government.	✓	✓	I
	Mammography is recommended as a breast cancer screening by the government.	✓	✓	C
	**An annual examination from age 20 years consisting of mammography and breast ultrasound is recommended as a breast cancer screening by the government.**	✓	✓	I
	A pap test of the cervix is recommended as a cervical cancer screening by the government.	✓	✓	C
	Whole-body PET is recommended as a cancer screening by the government.	✓	✓	I

	A fecal occult blood test is to check for blood in the stool by colonoscopy.	✓	✓	I
	A fecal occult blood test is to check for blood in the stool you scraped with a brush.	✓	✓	C
	You should undergo colorectal cancer screening by colonoscopy every year because its safety is established.	✓	✓	I
	**The benefit of cancer screening is early detection and early intervention to suspected lesions.**	✓	✓	C
	**Cancer screening can detect any small lesions.**	✓	✓	I
	All cancer screenings should be undergone at a younger age, in your 20's and 30's.	✓	✓	I
	You should continue to undergo cancer screenings throughout your life.	✓	✓	I
	The goal of cancer screenings is to reduce deaths from cancer.	✓	✓	C
	The goal of cancer screenings is to find as many cancers as possible.	✓	✓	I
	The greatest disadvantage of cancer screening is the side effects of the examinations, such as pain and radiation exposures.	✓	✓	I
	You should be aware of overdiagnosis by cancer screenings.	✓	✓	C
	Elevated levels of tumor markers mean that there is cancer somewhere in your body.	✓	✓	I
Treatment	Cancer treatments consist of surgery, radiotherapy, and chemotherapy.	✓	✓	C
	**The three main cancer treatments consist of surgery, chemotherapy, and immunotherapy.**	✓	✓	I
	Hormone therapy consists of cancer treatment.	✓	✓	C
	Palliative treatment consists of important cancer treatment.	✓	✓	C
	Surgery is needed for a complete cure for cancer.	✓	✓	I
	Chemotherapy always causes nausea and vomiting.	✓	✓	I
	You don't feel any pain during radiotherapy.	✓	✓	C
	Immunotherapy is proven effective for any cancer.	✓	✓	I
	Palliative treatments are used only in the terminal stages of cancer.	✓	✓	C
	Palliative treatments relieve pain but don't prolong life.	✓	✓	I
	The therapeutic effects of surgery and radiotherapy on some cancers are comparable.	✓	✓	C

	The side effects of chemotherapy are entirely different depending on the type.	✓	✓	C
	You should seek careful confirmation when you receive alternative medicine. (revised for 2nd)		✓	C
	**Cancer is not completely cured by radiotherapy.**	✓	✓	I
	The doctor should decide the treatment of your cancer, even if you have some candidates for treatments.	✓	✓	I
	It's enough for patients to obey what their doctors decide, though patients are not experts.	✓	✓	I
	**Patients should discuss with their doctors when making decisions about their cancer treatments.**	✓	✓	C
Coping with disease	**Second opinions are not recommended in oncological fields because consulting for opinions requires time and cost.**	✓	✓	I
	Cancer patient support centers are available when you are diagnosed or suspected of cancer.	✓	✓	C
	Cancer patient groups and support groups are available when you are diagnosed or suspected of cancer.	✓	✓	C
	**Occupational health care services are available to every employee when they are diagnosed or suspected of cancer.**	✓	✓	C
	The cancer support services provide psychological support.	✓		C
	The cancer support services provide financial support.	✓		C
	The cancer support services provide organizational support.	✓		C
	The cancer support services provide spiritual support.	✓		C
	The cancer support services provide family support.	✓		C
	**Medical information is private and cannot be generally divulged to others without permission.**	✓	✓	C
Information	**Cancer information from families is more trustworthy than from doctors because families are more kind.**	✓	✓	I
	**Cancer information from medical guidelines is as trustworthy as from TVs and radios because each has its advantage.**	✓	✓	I
	The medical information published in weekly magazines is more reliable because of their novelties than the information published by the National Cancer Center.	✓	✓	I

Abbreviations: JCIQ, Japanese Cancer Intelligence Quotient, JCIQ-L, Japanese Cancer Intelligence Quotient-Literacy; JCIQ-K, Japanese Cancer Intelligence Quotient-Knowledge; C, Correct; I, Incorrect; BMI, body mass index

Final items of the JCIQ are shown in bold.

✓ represented candidate items in each step.
